# Atypical presentation of classic Kaposi sarcoma in circumcised penis presenting as an ulcerative nodule with human herpesvirus 8 (HHV8) positivity and successfully treated with only local excision

**DOI:** 10.1186/s13027-019-0261-6

**Published:** 2019-12-02

**Authors:** Abdulaziz Alamri, B. K. Adiga

**Affiliations:** 1 College of Medicine, King Khalid University, Abha, Kingdom of Saudi Arabia; 2 Department of Pathology, College of Medicine, King Khalid University, Abha, Kingdom of Saudi Arabia

**Keywords:** Kaposi sarcoma, Penile nodule, HHV-8

## Abstract

**Introduction:**

Although the Kaposi sarcoma (KS) is one of the AIDS defining entity and seen in almost one third of HIV infected patients with low CD4 cell counts, it is not uncommon in HIV seronegative persons, but genital KS is rare, particularly in people without risk factors for HIV infection. Isolated penile KS is an unusual manifestation, especially as solitary nodule with ulceration, in HIV seronegative patient.

**Case presentation:**

We report such a case of Kaposi sarcoma showing HHV-8 positivity in an elderly male Arabian patient with a delay in prompt diagnosis, but treated successfully with 3 3 years follow-up after limited local surgical excision.

**Conclusion:**

The general practitioners, venereologists and urologists should think of KS as a possibility in such lesion and consider early biopsy.

## Introduction

Though Moritz Kaposi in 1872 described 5 cases of aggressive forms of sarcoma with mortality within 3 years, the incidence rate of Kaposi sarcoma increased with the discovery of HIV during early 1980s and its association with this vascular tumor. But the real causation of Kaposi sarcoma has been linked with HHV-8 in 1994 [1]. As the Kaposi sarcoma is known to be a diverse entity clinically, histopathologically & prognostically with its various forms, early diagnosis and roper treatment is of utmost significance in the management. Our patient of classic KS with rare presentation as isolated penile ulcerative nodule who had initial delay in diagnosis, but ultimately received proper management with good response.

### Case report

A 63 year old man was referred from a primary health center with a penile lesion of 8 months duration. The lesion started as a small, pruritic, slightly thickened brown discoloration on glans penis, for which a topical corticosteroid cream was prescribed with a clinical suspicion as lichen planus. But it continued to grow to reach the present size with recent surface ulceration.

There was no history of any systemic disease except he has been on diet for glucose intolerance.

**Fig. 1:**
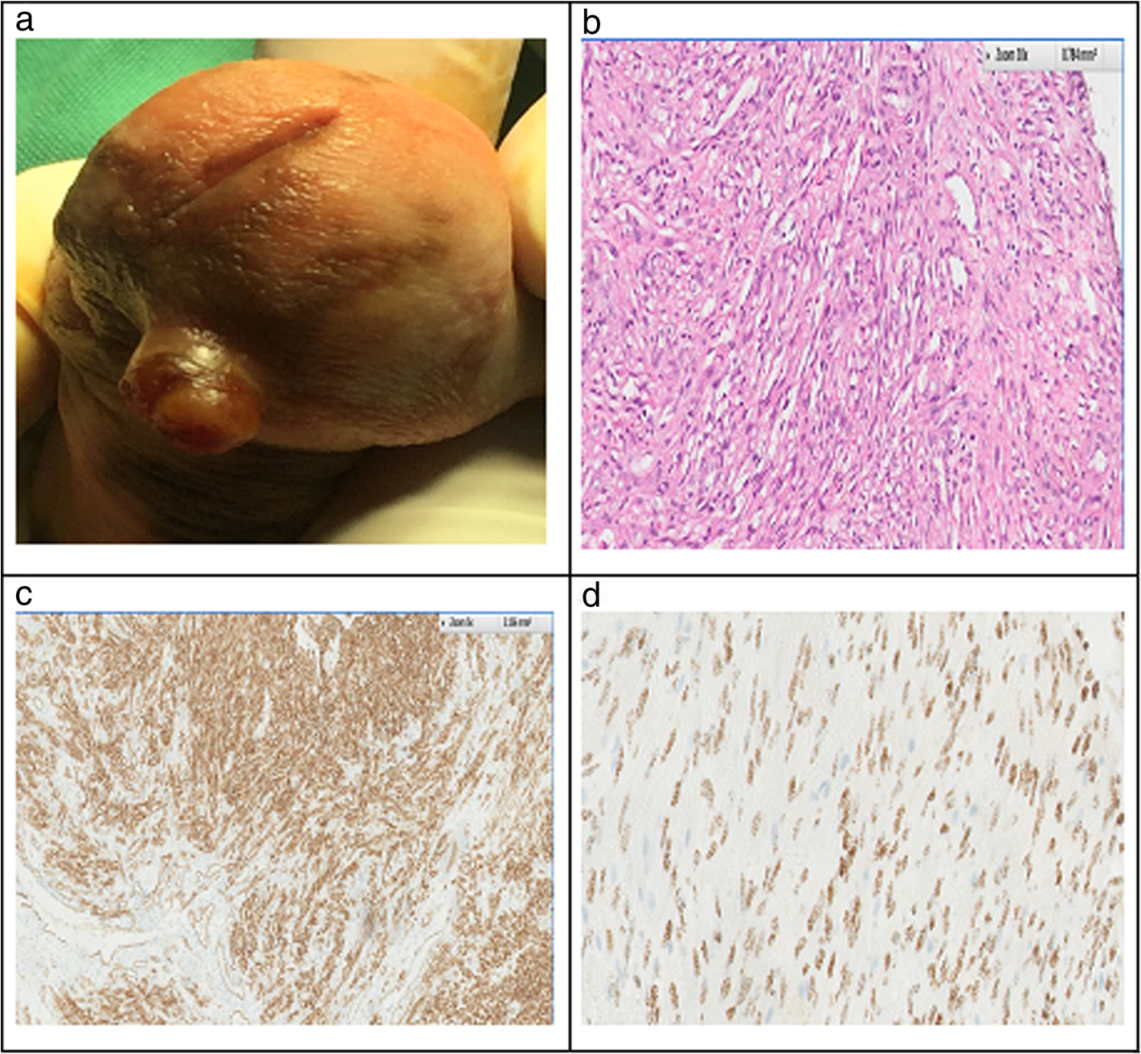
**a** Clinical picture of penile ulcerative nodule on glans penis. **b** Photomicrograph — H&E stain: Bundles of proliferating spindle cells wild mild atypia & foci of sprinkled RBC’s. **c** CD34 Immunostain: Cytoplasmic staining of spindle cells as well as endothelial cells of capillaries indicating spindles cells of endothelial origin. **d** HHV-8 immunostain: Demonstrates nuclear positivity to HHV-8 LNA-1 confirming Kaposi’s sarcoma

No history of exposure to risk factors of sexually transmitted diseases obtained. There was history of mild intermittent voiding and post void dribbling. No history of fever, weight loss or any other skin diseases. Local examination revealed an ulcerated firm, reddish brown, slightly tender nodule of 8 mm. on glans penis near corona on the ventral aspect of the penis (Fig. 1a).

No other similar lesions seen in genitalia. No inguinal lymphadenopathy noted. Dermatologic examination also did not reveal any skin or oral lesions.

The laboratory investigations for CBC, serum creatinine, serum PSA and complete urine analysis including urine culture did not show abnormal result. Swab culture from ulcer was negative. The blood HbA1c was 5.8%. The serological tests for HIV, HBsAg and RPR were negative. The pelvic ultrasound showed moderately enlarged prostate (size of 45 g.), but otherwise normal study.

After a signed consent, the patient underwent an excisional biopsy with the clinical impression of pyogenic granuloma. The initial histopathology report revealed atypical spindle cell proliferation with clear resection margins and advised immunohistochemical studies for final diagnosis. The immunostains revealed the spindle cells were immunoreactive for CD31, CD34 &HHV8 LANA-1 and non-reactive for SMA. (Fig. 1b, c, d); consistent with Kaposi sarcoma.

On consultation, uro-oncologist advised close clinical follow - up for any recurrence. No local recurrence or systemic lesions observed during last three years of follow up.

## Discussion

Kaposi sarcoma is considered as a borderline malignant tumor derived from lymphatic endothelial cells and is common in advanced HIV infection with low CD4 cell count. There are 5 categories which include classic, endemic, epidemic, iatrogenic and non-epidemic types [2].

Though the common denominator is the underlying immunosuppression in majority of patients, it is known to be caused by HHV-8 in almost all cases, even in immunocompetent persons [3]. The genitalia is involved in about 20% of HIV associated KS, usually as part of systemic disease, but very rare in non- HIV associated classic KS [4, 5]. The classic Kaposi sarcoma usually presents as sporadic cases, more commonly in Mediterranean & East European population, in elderly HIV seronegative people with multiple brownish non-pruritic skin patches, plaques and nodules, usually affecting lower limbs [6], but rare to present as isolated genital solitary lesion, that too in Arabic patient as in our case. Linker et al. reported first 4 cases of primary penile KS [7]. Upto 2003, 19 cases of primary penile KS documented, including only 2 cases manifested as solitary ulcerated nodule, but without HIV status [8]. Micali et al. identified only 12 cases of primary penile KS in non-HIV patients during his review of literature. But first case of solitary penile KS with HHV8 positivity in HIV seronegative patient was published by Morelli et al. [9].

Many cases of penile KS presents as multiple macules or papules or nodules confining only to.

penis or genital area, but more commonly as part of cutaneous lesions elsewhere.

The first genital Kaposi sarcoma in Saudi Arabia was reported in 1994, with multiple papules on penis and scrotum [10]. In 2016 two cases of primary solitary penile KS were published from Dhahran region of Saudi Arabia [11]. Our patient also had only solitary penile lesion, but ulcerated mimicking pyogenic granuloma. The histological pattern of Kaposi sarcomaalso vary mimicking pyogenic granuloma, hemangioma, lymphangioma, acroangiodermatitis,immature scar and spindle cell tumor’s like leiomyoma, angiofibroma, myofibroma, fibrous histiocytoma and angiosarcoma. Though the CD31 & CD34 immuno-stains recognize the lesion as endothelial origin, the HHV8 antigen immuno- reactivity in the tissue is diagnostic for Kaposi sarcoma [12]. Still the latter immunostains may not be available in many laboratories. There was no obvious sign of any systemic immunosuppression. But the patient received prolonged local steroid application which might be the predisposing factor for HHV8 infection or reactivation of prior infection. Though there was denial about extra marital relationship, sexual transmission of HHV8 virus cannot be excluded. HHV8 LANA-1 antigen is known to bind to P53 and Rb proteins to alter transcriptional activity of genes involved in cell cycle and apoptosis 3. HIV associated KS is known to be clinically aggressive compared to the more indolent course of classic KS [13]. In our patient, the lesion was slowly progressive but later developed ulcer and then referred to tertiary medical center. The final diagnosis of KS was made on excision biopsy as tumor cells were positive for HHV8, LANA-1, CD34 & CD31.

In one study, 13 patients of penile KS were managed with local excision, but 4 of them showed local recurrence [8]. Our patient had uneventful recovery with proper healing and no local or systemic recurrence of the malignancy observed during 3 years of regular follow - up.

## C**onclusion**

The sporadic Kaposi sarcoma can present in an unusual manner in rare sites without any known risk factors. The atypical features in the current case are KS manifesting as only penile single ulcerative nodule resembling pyogenic granuloma and demonstrating HHV8 LANA-1 immunoreactivity in HIV negative elderly person, with no recurrence after proper excision. The general practitioners, venereologists and urologists should think of KS as a possibility in such lesion and consider early biopsy. The proper early treatment and regular follow -up will facilitate better health care.

## Data Availability

The data used (lab reports and clinical findings) during the current study are available from the corresponding author on reasonable request.
